# Editorial perspective of the Research Topic “Deciphering serotonin's role in neurodevelopment”

**DOI:** 10.3389/fncel.2013.00212

**Published:** 2013-11-18

**Authors:** Judith R. Homberg, Sharon M. Kolk, Dirk Schubert

**Affiliations:** ^1^Department of Cognitive Neuroscience, Donders Institute for Brain, Cognition and Behavior, Radboud University Nijmegen Medical CentreNijmegen, Netherlands; ^2^Department of Molecular Animal Physiology, Donders Institute for Brain, Cognition and Behavior, Radboud University NijmegenNijmegen, Netherlands

**Keywords:** serotonin, neurodevelopment, placental serotonin, sensory system, prefrontal cortex, raphe nuclei, cortical integrity, autism

Serotonin is implicated in many, if not all, psychiatric disorders and is therefore the most studied neurotransmitter in our brain. Nevertheless, the developing serotonergic system and especially its role during brain maturation are still poorly understood. The role of serotonin in psychiatric conditions like anxiety, depression, and autism is either investigated in advanced stages of the disorder or in the context of selective serotonin reuptake inhibitor (SSRI) treatment. However, there is ample evidence for serotonin playing a crucial role in the early development of the nervous system, and that this role is different from the function of serotonin in the mature brain.

In this Research Topic entitled “Deciphering serotonin's role in neurodevelopment” we, together with leaders in the field, have brought together the most recent insights in serotonin's diverse roles especially during development, by means of both reviews and new empirical data. (Smidt and van Hooft, 2013) provide an overview of the development of the serotonergic system in rodents. Serotonergic neurons are born at embryonic day 10.5 caudal to the mid-hindbrain border (the isthmus). A plethora of transcription factors are temporally and spatially expressed here which determine the fate of the newborn neurons and their serotonergic phenotype. Yet, local synthesis within the raphe nuclei is not the only source for serotonin during embryonic brain development. As reviewed by Velasquez et al. (2013) the placenta is additionally involved in the synthesis of serotonin using maternally derived tryptophan. This placental source of serotonin may be a critical link between early genetic and environmental perturbations and their impact on brain maturation, including the development of the serotonergic system itself. As such, in the paper by Witteveen et al. (2013) the outgrowth of serotonergic neurons from the rostral raphe cluster to the medial prefrontal cortex (mPFC) is investigated as a function of genetic variance in the gene encoding the serotonin transporter (5-HTT) using dorsal/median raphe and prefrontal explants. It was found that whereas the dorsal raphe serotonergic outgrowing neurites remained unaffected by the loss of 5-HTT, the median raphe serotonergic neurites switched from a strong repulsive toward an attractive interaction when cocultured with the mPFC. As a result, the mPFC of 5-HTT deficient rats may receive more serotonergic innervation from the median raphe nucleus compared to wild-type rats. Furthermore, it was shown that the number of Satb2-positive callosal projection neurons was reduced in absence of the 5-HTT. Besides the development of the raphe- prefrontal network formation also the anatomical and physiological properties of the somatosensory system is affected by 5-HTT ablation. Miceli et al. (2013) report that thalamocortical afferents (TCA's) innervating their main target structures in layer 4 of the somatosensory cortex, the “barrels” representing the whiskers, are more diffuse and less topologically organized in absence of the 5-HTT. Accordingly, the barrel cortex pattern, although clearly present in 5-HTT deficient rats, was more diffuse with smaller barrels and increased inter-barrel widths. It is well possible that these extensive structural alterations in the topological organization affect somatosensory (whisker-mediated) perceptions. Intriguingly, these perceptions are indeed reduced in 5-HTT knockout mice [reviewed by Kinast et al. (2013)]. The somatosensory system is not the only sensory system showing a dependency on serotonin during brain development, as was demonstrated by Zhang et al. (2013). The serotonergic raphe nuclear complex projects directly to the olfactory bulb and olfactory performance is known to depend strongly on serotonin. The authors found that neonatal SSRI application caused a gender specific reduction in the 5-HTT expressing fibers that innervate the olfactory bulb in rats.

Serotonin can act through one of the 15 identified 5-HT receptor subtypes. Besides the 5-HT_1B_ receptor, the 5-HT_3_, and 5-HT_6_ receptors may play specific roles in the serotonin-mediated neurodevelopmental processes as nicely reviewed by Vitalis et al. (2013). Different aspects of cortical construction such as neuronal migration or dendritic differentiation are steered through the 5-HT_3A_ and the 5-HT_6_, receptor, respectively. Indeed, as reviewed by Engel et al. (2013) 5-HT_3_ receptors expressed on cortical interneurons and Cajal-Retzius cells regulate the morphology, positioning, and connectivity of the local microcircuitry during late embryogenesis. As the authors suggest, the 5-HT_3_ receptor may play an important role in autism, given that mice lacking the 5-HT_3_ receptor show social impairments and hypercomplexity of cortical layer 2/3 as is characteristic for autism. Furthermore, the 5HT_3_ receptor is a likely target of prenatal SSRI effects as neatly described by Olivier et al. (2013).

As reviewed by Kinast et al. (2013) the cognitive (PFC-dependent) and somatosensory phenotypes observed in 5-HTT knockout rodents as well as human subjects carrying the low activity variant of the serotonin transporter linked polymorphic region (5-HTTLPR) strikingly resemble those seen in autistic patients, rats prenatally treated with VPA (rat model for autism), and human and rodent subjects prenatally exposed to SSRIs (see also Olivier et al., 2013) (Figure 1). However, these commonalities may be dependent on maternal depression, as executive function got worse in children being homozygous for the 5-HTTLPR long allele when the mother was depressed, whereas children prenatally exposed to SSRIs and carrying the 5-HTTLPR short allele were insensitive to maternal depression (Weikum et al., 2013). Nonetheless, loss of 5-HTT, prenatal SSRI exposure and autism may be interconnected by both showing a reduction in callosal-dependent intercortical connectivity, which—together with the finding of Witteveen et al. (2013) that callosal projection neurons seem to be altered in 5-HTT knockout rats—raise the possibility that serotonin affects the identity of projection neurons. Early serotonergic innervations may control laminar and cellular identities of cortical areas involved in complex behavior, possibly by acting on the reelin release by Cajal Retzius cells through the 5-HT_3_ receptor. The results presented in this Research Topic demonstrate the crucial role of serotonin in neurodevelopment and thereby reveals itself as a key player in the onset of neuropsychiatric disorders like anxiety, depression, and autism.

**Figure 1:**
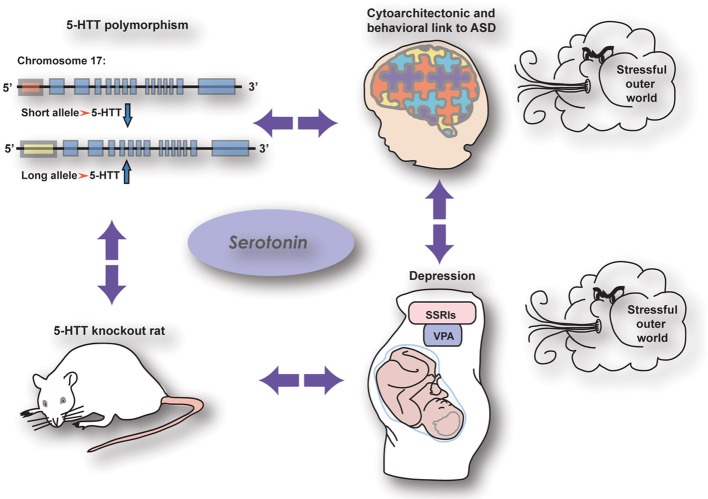
**The relationship between genetic and pharmacological manipulations affecting the serotonin system showing similar effects on brain wiring and behaviour (from Kinast et al., 2013), see below**.

## References

[B1] Engel M. Smidt M. P. van Hooft J. A. (2013). The serotonin 5-HT3 receptor: a novel neurodevelopmental target. Front. Cell. Neurosci. 7:76 10.3389/fncel.2013.00076 23761731 PMC3669892

[B2] Kinast K. Peeters D. Kolk S. M. Schubert D. Homberg J. R. (2013). Genetic and pharmacological manipulations of the serotonergic system in early life: neurodevelopmental underpinnings of autism-related behavior. Front. Cell. Neurosci. 7:72 10.3389/fncel.2013.00072 23781172 PMC3679613

[B3] Miceli S. Negwer M. van Eijs F. Kalkhoven C. van Lierop I. Homberg J. (2013). High serotonin levels during brain development alter the structural input-output connectivity of neural networks in the rat somatosensory layer IV. Front. Cell. Neurosci. 7:88 10.3389/fncel.2013.00088 23761736 PMC3675331

[B4] Olivier J. D. Akerud H. Kaihola H. Pawluski J. L. Skalkidou A. Högberg U. (2013). The effects of maternal depression and maternal selective serotonin reuptake inhibitor exposure on offspring. Front. Cell. Neurosci. 7:73 10.3389/fncel.2013.00073 23734100 PMC3659337

[B4a] Smidt M. P. van Hooft J. A. (2013) Subset specification of central serotonergic neurons. Front. Cell. Neurosci. 7:200 10.3389/fncel.2013.00200 24198761 PMC3813900

[B5] Velasquez J. C. Goeden N. Bonnin A. (2013). Placental serotonin: implications for the developmental effects of SSRIs and maternal depression. Front. Cell. Neurosci. 7:47 10.3389/fncel.2013.00047 23630464 PMC3632750

[B6] Vitalis T. Ansorge M. S. Dayer A. G. (2013). Serotonin homeostasis and serotonin receptors as actors of cortical construction: special attention to the 5-HT3A and 5-HT6 receptor subtypes. Front. Cell. Neurosci. 7:93 10.3389/fncel.2013.00093 23801939 PMC3686152

[B7] Weikum W. M. Brain U. Chau C. M. Grunau R. E. Boyce W. T. Diamond A. (2013). Prenatal serotonin reuptake inhibitor (SRI) antidepressant exposure and serotonin transporter promoter genotype (SLC6A4) influence executive functions at 6 years of age. Front. Cell. Neurosci. 7:180 10.3389/fncel.2013.00180 24130516 PMC3795328

[B8] Witteveen J. S. Middelman A. van Hulten J. A. Martens G. J. Homberg J. R. Kolk S. M. (2013). Lack of serotonin reuptake during brain development alters rostral raphe-prefrontal network formation. Front. Cell. Neurosci. 7:143 10.3389/fncel.2013.00143 24109430 PMC3790074

[B9] Zhang J. Dennis K. A. Darling R. D. Alzghoul L. Paul I. A. Simpson K. L. (2013). Neonatal citalopram exposure decreases serotonergic fiber density in the olfactory bulb of male but not female adult rats. Front. Cell. Neurosci. 7:67 10.3389/fncel.2013.00067 23675318 PMC3650517

